# 1-[(Diethyl­amino­carbon­yl)meth­yl]-2-[hydr­oxy(6-methoxy­quinolin-4-yl)meth­yl]-5-vinyl-1-azoniabicyclo­[2.2.2]octane chloride monohydrate

**DOI:** 10.1107/S1600536807068444

**Published:** 2008-01-25

**Authors:** Li-Ping Zhang, Lin-Juan Wei, Ming-Qing Chen

**Affiliations:** a School of Chemical and Materials Engineering, Jiangnan University, 1800 Lihu Road, Wuxi 214122, Jiangsu, People’s Republic of China

## Abstract

In the title compound, C_26_H_36_N_3_O_3_^+^·Cl^−^·H_2_O, the mol­ecular structure of the cation is stabilized by a number of C—H⋯O intra­molecular inter­actions. In the crystal structure, O—H⋯Cl and C—H⋯Cl hydrogen bonds link the ions into a ribbon-like structure along the *a* axis.

## Related literature

For related structures, see: Oleksyn *et al.* (1979 ▶); Zhang *et al.* (2006 ▶).
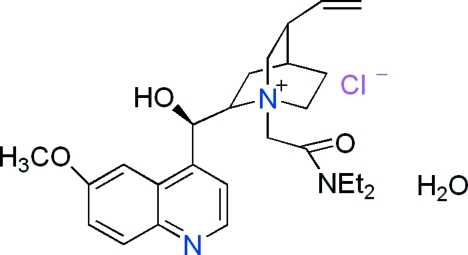


## Experimental

### 

#### Crystal data

C_26_H_36_N_3_O_3_^+^·Cl^−^·H_2_O
*M* *_r_* = 492.04
Orthorhombic, 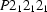

*a* = 8.2213 (12) Å
*b* = 17.441 (3) Å
*c* = 18.161 (3) Å
*V* = 2604.0 (7) Å^3^
*Z* = 4
Mo *K*α radiation
μ = 0.18 mm^−1^
*T* = 292 K
0.24 × 0.20 × 0.16 mm

#### Data collection

Bruker SMART CCD area-detector diffractometer
Absorption correction: multi-scan (*SADABS*; Sheldrick, 1996 ▶) *T*_min_ = 0.938, *T*_max_ = 0.973
15334 measured reflections
5361 independent reflections
3043 reflections with *I* > 2σ(*I*)
*R*_int_ = 0.061

#### Refinement

*R*[*F* ^2^ > 2σ(*F* ^2^)] = 0.048
*wR*(*F* ^2^) = 0.111
*S* = 0.99
5361 reflections
311 parameters
H-atom parameters constrained
Δρ_max_ = 0.15 e Å^−3^
Δρ_min_ = −0.18 e Å^−3^
Absolute structure: Flack (1983 ▶), with 2317 Friedel pairs
Flack parameter: 0.14 (9)

### 

Data collection: *SMART* (Bruker, 1998 ▶); cell refinement: *SAINT* (Bruker, 1999 ▶); data reduction: *SAINT*; program(s) used to solve structure: *SHELXS97* (Sheldrick, 2008 ▶); program(s) used to refine structure: *SHELXL97* (Sheldrick, 2008 ▶); molecular graphics: *SHELXTL* (Bruker, 1999 ▶); software used to prepare material for publication: *SHELXL97*.

## Supplementary Material

Crystal structure: contains datablocks global, I. DOI: 10.1107/S1600536807068444/ci2541sup1.cif

Structure factors: contains datablocks I. DOI: 10.1107/S1600536807068444/ci2541Isup2.hkl

Additional supplementary materials:  crystallographic information; 3D view; checkCIF report

## Figures and Tables

**Table 1:** Hydrogen-bond geometry (Å, °)

*D*—H⋯*A*	*D*—H	H⋯*A*	*D*⋯*A*	*D*—H⋯*A*
O2—H2⋯Cl1	0.82	2.25	3.038 (2)	161
C19—H19*A*⋯Cl1^i^	0.97	2.70	3.580 (4)	150
C20—H20*B*⋯O2	0.97	2.33	3.001 (4)	126
C21—H21*B*⋯Cl1	0.97	2.76	3.650 (3)	152
C21—H21*B*⋯O2	0.97	2.58	3.169 (4)	119
O4⋯Cl1^ii^			3.141 (4)	
O4⋯Cl1^iii^			3.214 (4)	

Symmetry codes: (i) 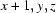; (ii) 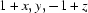; (iii) 
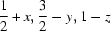
.

**Fig. 1.**
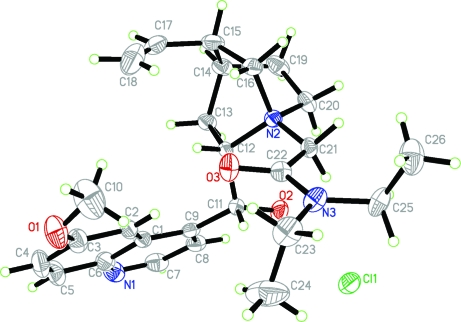
The asymmetric unit of the title compound, showing 30% probability displacement ellipsoids. Water molecule has been omitted for clarity.

**Fig. 2.**
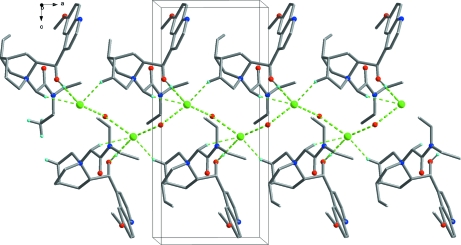
The molecular packing of the title compound, viewed along the b axis. H atoms not involved in hydrogen bonding have been omitted.

## supplementary crystallographic information

### Comment

In the title compound (Fig. 1), the quinoline ring system is planar with a maximum deviation of 0.026 (3) Å for atom C8. Bond lengths and angles are comparable to those observed in a related cinchonine structure (Oleksyn *et al.*, 1979) but the molecules differ slightly in the relative orientations of azoniabicyclo[2.2.2]octane and quinoline units.

The structure of cation is stabilized by a number of C—H···O intramolecular interactions. In the crystal structure O—H···Cl, C—H···Cl and O_w_···Cl interactions link the ions into a ribbon along the *a* axis (Fig.2). Similar packing arrangement is found in the structure of a related cinchonine quaternary salt (Zhang *et al.*, 2006).

### Experimental

The title compound was prepared by the reaction of 2-chloro-*N*,*N*-diethylacetamide (3 mmol) with quinine (2 mmol) in acetone (5 ml) refluxed for 5 h under a N_2_ atmosphere. The resulting precipitate was isolated by filtration, washed, dried, and recrystallized from Et_2_O and CH_2_Cl_2_ (7:1). Single crystals suitable for X-ray diffraction study were obtained from CH_2_Cl_2_ by slow evaporation at room temperature.

### Refinement

H atoms were placed in geometrically idealized positions and constrained to ride on their parent atoms, with O—H = 0.82 Å, C—H = 0.93–0.98 Å, and *U*_iso_(H) = 1.5*U*_eq_(O_OH_, C_CH_3) or 1.2*U*_eq_(C). Each methyl group was allowed to rotate freely about its C—C bond. H-atoms bound to the oxygen atom of the water molecule could not be located from difference Fourier maps.

### Crystal data

**Table:**

C_26_H_36_N_3_O_3_^+^·Cl^–^·H_2_O	*F*_000_ = 1056
*M**_r_* = 492.04	*D*_x_ = 1.255 Mg m^−^^3^
Orthorhombic, *P*2_1_2_1_2_1_	Mo *K*α radiation λ = 0.71073 Å
Hall symbol: P 2ac 2ab	Cell parameters from 2754 reflections
*a* = 8.2213 (12) Å	θ = 2.3–21.7º
*b* = 17.441 (3) Å	µ = 0.18 mm^−^^1^
*c* = 18.161 (3) Å	*T* = 292 K
*V* = 2604.0 (7) Å^3^	Block, colourless
*Z* = 4	0.24 × 0.20 × 0.16 mm

### Data collection

**Table:**

Bruker SMART CCD area-detector diffractometer	5361 independent reflections
Radiation source: fine-focus sealed tube	3043 reflections with *I* > 2σ(*I*)
Monochromator: graphite	*R*_int_ = 0.062
*T* = 292 K	θ_max_ = 26.5º
φ and ω scans	θ_min_ = 1.6º
Absorption correction: multi-scan(SADABS; Sheldrick, 1996)	*h* = −6→10
*T*_min_ = 0.938, *T*_max_ = 0.973	*k* = −21→21
15334 measured reflections	*l* = −19→22

### Refinement

**Table:**

Refinement on *F*^2^	Hydrogen site location: inferred from neighbouring sites
Least-squares matrix: full	H-atom parameters constrained
*R*[*F*^2^ > 2σ(*F*^2^)] = 0.048	*w* = 1/[σ^2^(*F*_o_^2^) + (0.0381*P*)^2^ + 0.4431*P*] where *P* = (*F*_o_^2^ + 2*F*_c_^2^)/3
*wR*(*F*^2^) = 0.111	(Δ/σ)_max_ = 0.001
*S* = 1.00	Δρ_max_ = 0.15 e Å^−^^3^
5361 reflections	Δρ_min_ = −0.18 e Å^−^^3^
311 parameters	Extinction correction: none
Primary atom site location: structure-invariant direct methods	Absolute structure: Flack (1983), 2317 Friedel pairs
Secondary atom site location: difference Fourier map	Flack parameter: 0.14 (9)

### Special details

**Table:**

Geometry. All e.s.d.'s (except the e.s.d. in the dihedral angle between two l.s. planes) are estimated using the full covariance matrix. The cell e.s.d.'s are taken into account individually in the estimation of e.s.d.'s in distances, angles and torsion angles; correlations between e.s.d.'s in cell parameters are only used when they are defined by crystal symmetry. An approximate (isotropic) treatment of cell e.s.d.'s is used for estimating e.s.d.'s involving l.s. planes.
Refinement. Refinement of *F*^2^ against ALL reflections. The weighted *R*-factor *wR* and goodness of fit *S* are based on *F*^2^, conventional *R*-factors *R* are based on *F*, with *F* set to zero for negative *F*^2^. The threshold expression of *F*^2^ > σ(*F*^2^) is used only for calculating *R*-factors(gt) *etc*. and is not relevant to the choice of reflections for refinement. *R*-factors based on *F*^2^ are statistically about twice as large as those based on *F*, and *R*- factors based on ALL data will be even larger.

### Fractional atomic coordinates and isotropic or equivalent isotropic displacement parameters (Å^2^)

**Table:**

	*x*	*y*	*z*	*U*_iso_*/*U*_eq_	
O1	0.3225 (4)	0.93582 (15)	0.56437 (14)	0.0753 (8)	
O2	0.4216 (3)	0.66267 (12)	0.82251 (11)	0.0479 (6)	
H2	0.3525	0.6737	0.8533	0.072*	
O3	0.5222 (3)	0.90203 (12)	0.78329 (11)	0.0526 (6)	
N2	0.6959 (3)	0.76896 (13)	0.82281 (11)	0.0354 (6)	
N3	0.4329 (4)	0.92548 (14)	0.89798 (14)	0.0464 (7)	
C1	0.3147 (4)	0.73809 (18)	0.63577 (14)	0.0376 (7)	
C2	0.3419 (4)	0.81809 (18)	0.63353 (16)	0.0410 (8)	
H2A	0.3878	0.8426	0.6739	0.049*	
C3	0.3012 (5)	0.8596 (2)	0.57254 (18)	0.0539 (9)	
C4	0.2293 (5)	0.8234 (2)	0.51162 (18)	0.0663 (12)	
H4	0.2016	0.8523	0.4705	0.080*	
C5	0.2000 (5)	0.7482 (3)	0.51186 (17)	0.0637 (10)	
H5	0.1515	0.7254	0.4711	0.076*	
C6	0.2417 (4)	0.7029 (2)	0.57315 (17)	0.0470 (9)	
N1	0.2080 (4)	0.62659 (18)	0.56833 (16)	0.0588 (8)	
C7	0.2426 (4)	0.5857 (2)	0.6256 (2)	0.0548 (10)	
H7	0.2206	0.5335	0.6232	0.066*	
C8	0.3111 (4)	0.61412 (19)	0.69128 (17)	0.0474 (8)	
H8	0.3299	0.5814	0.7308	0.057*	
C9	0.3497 (4)	0.69032 (18)	0.69654 (15)	0.0363 (8)	
C10	0.4145 (7)	0.9754 (2)	0.6174 (3)	0.1019 (17)	
H10A	0.5159	0.9494	0.6250	0.153*	
H10B	0.3552	0.9773	0.6629	0.153*	
H10C	0.4351	1.0267	0.6005	0.153*	
C11	0.4261 (4)	0.72014 (16)	0.76742 (14)	0.0328 (7)	
H11	0.3644	0.7648	0.7845	0.039*	
C12	0.6021 (3)	0.74435 (18)	0.75294 (13)	0.0320 (7)	
H12	0.5983	0.7890	0.7202	0.038*	
C13	0.7026 (4)	0.68341 (19)	0.71416 (16)	0.0414 (8)	
H13A	0.6918	0.6892	0.6613	0.050*	
H13B	0.6632	0.6329	0.7276	0.050*	
C14	0.8815 (4)	0.6912 (2)	0.73606 (18)	0.0486 (9)	
H14	0.9506	0.6614	0.7027	0.058*	
C15	0.9306 (4)	0.7755 (2)	0.73507 (17)	0.0527 (9)	
H15	1.0470	0.7775	0.7466	0.063*	
C16	0.8411 (4)	0.81627 (19)	0.79803 (16)	0.0440 (8)	
H16A	0.9148	0.8237	0.8391	0.053*	
H16B	0.8045	0.8663	0.7815	0.053*	
C19	0.8954 (5)	0.6608 (2)	0.81431 (18)	0.0571 (10)	
H19A	1.0031	0.6715	0.8336	0.069*	
H19B	0.8793	0.6057	0.8146	0.069*	
C20	0.7671 (4)	0.69938 (17)	0.86258 (16)	0.0464 (9)	
H20A	0.8160	0.7155	0.9087	0.056*	
H20B	0.6810	0.6631	0.8737	0.056*	
C17	0.9092 (5)	0.8107 (3)	0.6606 (2)	0.0611 (11)	
H17	0.9496	0.7822	0.6214	0.073*	
C18	0.8428 (6)	0.8751 (3)	0.6433 (2)	0.0897 (15)	
H18A	0.7999	0.9065	0.6799	0.108*	
H18B	0.8377	0.8902	0.5943	0.108*	
C21	0.5994 (4)	0.81309 (17)	0.87826 (14)	0.0370 (7)	
H21A	0.6714	0.8280	0.9181	0.044*	
H21B	0.5172	0.7794	0.8988	0.044*	
C22	0.5158 (4)	0.88459 (17)	0.84909 (17)	0.0388 (8)	
C23	0.3385 (5)	0.9913 (2)	0.8714 (2)	0.0639 (11)	
H23A	0.3963	1.0152	0.8309	0.077*	
H23B	0.3297	1.0288	0.9107	0.077*	
C24	0.1710 (6)	0.9696 (3)	0.8462 (3)	0.116 (2)	
H24A	0.1116	0.9479	0.8866	0.174*	
H24B	0.1788	0.9327	0.8071	0.174*	
H24C	0.1154	1.0145	0.8287	0.174*	
C25	0.4319 (5)	0.9119 (2)	0.97795 (17)	0.0571 (10)	
H25A	0.4590	0.8587	0.9874	0.069*	
H25B	0.3233	0.9210	0.9968	0.069*	
C26	0.5499 (6)	0.9624 (2)	1.0182 (2)	0.0776 (14)	
H26A	0.5298	1.0150	1.0056	0.116*	
H26B	0.6589	0.9489	1.0044	0.116*	
H26C	0.5365	0.9556	1.0703	0.116*	
Cl1	0.19903 (11)	0.74211 (6)	0.93207 (4)	0.0619 (3)	
O4	0.9536 (5)	0.8684 (2)	−0.01438 (18)	0.1224 (12)	

### Atomic displacement parameters (Å^2^)

**Table:**

	*U*^11^	*U*^22^	*U*^33^	*U*^12^	*U*^13^	*U*^23^
O1	0.102 (2)	0.0584 (17)	0.0655 (16)	0.0077 (16)	−0.0188 (19)	0.0180 (14)
O2	0.0541 (17)	0.0496 (14)	0.0399 (12)	−0.0007 (12)	0.0049 (12)	0.0114 (11)
O3	0.0734 (18)	0.0461 (14)	0.0383 (13)	0.0105 (13)	0.0025 (13)	0.0030 (11)
N2	0.0321 (14)	0.0401 (15)	0.0341 (12)	0.0020 (13)	0.0009 (13)	−0.0032 (11)
N3	0.055 (2)	0.0343 (16)	0.0500 (16)	0.0071 (15)	0.0124 (15)	−0.0005 (12)
C1	0.0311 (17)	0.048 (2)	0.0341 (15)	0.0033 (17)	0.0008 (15)	−0.0072 (15)
C2	0.040 (2)	0.049 (2)	0.0342 (17)	0.0088 (17)	−0.0035 (16)	0.0010 (15)
C3	0.060 (2)	0.055 (2)	0.046 (2)	0.012 (2)	−0.001 (2)	0.0022 (18)
C4	0.082 (3)	0.078 (3)	0.0381 (19)	0.016 (3)	−0.009 (2)	0.005 (2)
C5	0.073 (3)	0.080 (3)	0.0382 (18)	0.009 (3)	−0.015 (2)	−0.009 (2)
C6	0.044 (2)	0.055 (2)	0.0419 (19)	0.0060 (17)	−0.0009 (17)	−0.0078 (17)
N1	0.0565 (19)	0.065 (2)	0.0547 (18)	0.0069 (17)	−0.0075 (18)	−0.0142 (17)
C7	0.045 (2)	0.050 (2)	0.069 (2)	−0.0047 (18)	−0.001 (2)	−0.020 (2)
C8	0.0370 (19)	0.051 (2)	0.054 (2)	−0.0063 (18)	0.0021 (18)	−0.0017 (16)
C9	0.0263 (18)	0.0421 (19)	0.0403 (17)	−0.0032 (15)	0.0043 (15)	−0.0049 (15)
C10	0.146 (5)	0.049 (3)	0.111 (4)	−0.002 (3)	−0.038 (4)	0.019 (3)
C11	0.0324 (18)	0.0349 (17)	0.0310 (15)	0.0007 (14)	0.0006 (14)	0.0008 (13)
C12	0.0288 (16)	0.0425 (18)	0.0247 (13)	0.0041 (15)	−0.0030 (13)	−0.0017 (13)
C13	0.0324 (18)	0.051 (2)	0.0407 (17)	0.0089 (18)	−0.0004 (17)	−0.0122 (15)
C14	0.032 (2)	0.062 (2)	0.051 (2)	0.0098 (18)	−0.0014 (17)	−0.0197 (18)
C15	0.0279 (19)	0.073 (3)	0.057 (2)	−0.0010 (19)	0.0005 (18)	−0.0126 (19)
C16	0.034 (2)	0.053 (2)	0.0451 (18)	−0.0070 (17)	−0.0019 (17)	−0.0046 (16)
C19	0.048 (2)	0.058 (2)	0.065 (2)	0.0162 (19)	−0.016 (2)	−0.0141 (19)
C20	0.052 (2)	0.044 (2)	0.0426 (18)	0.0094 (17)	−0.0153 (18)	−0.0010 (15)
C17	0.043 (2)	0.086 (3)	0.054 (2)	−0.013 (2)	0.014 (2)	−0.004 (2)
C18	0.110 (4)	0.095 (4)	0.064 (3)	−0.020 (3)	0.022 (3)	0.009 (3)
C21	0.0422 (19)	0.0399 (18)	0.0288 (14)	0.0031 (16)	−0.0018 (15)	−0.0058 (14)
C22	0.040 (2)	0.0332 (18)	0.0427 (19)	−0.0022 (16)	−0.0007 (16)	−0.0042 (15)
C23	0.075 (3)	0.040 (2)	0.077 (3)	0.020 (2)	0.010 (2)	0.0034 (19)
C24	0.057 (3)	0.106 (4)	0.186 (6)	0.015 (3)	0.008 (4)	0.059 (4)
C25	0.076 (3)	0.051 (2)	0.0440 (19)	0.003 (2)	0.023 (2)	−0.0046 (17)
C26	0.117 (4)	0.061 (3)	0.055 (2)	−0.019 (3)	−0.001 (3)	−0.0084 (19)
Cl1	0.0504 (5)	0.0839 (7)	0.0515 (5)	−0.0123 (5)	0.0083 (5)	−0.0087 (5)
O4	0.128 (3)	0.127 (3)	0.113 (2)	−0.002 (2)	0.014 (2)	−0.004 (2)

### Geometric parameters (Å, °)

**Table:**

O1—C3	1.349 (4)	C13—C14	1.529 (4)
O1—C10	1.406 (5)	C13—H13A	0.97
O2—C11	1.417 (3)	C13—H13B	0.97
O2—H2	0.82	C14—C19	1.521 (5)
O3—C22	1.234 (3)	C14—C15	1.525 (5)
N2—C21	1.495 (3)	C14—H14	0.98
N2—C16	1.519 (4)	C15—C17	1.496 (5)
N2—C20	1.529 (4)	C15—C16	1.535 (4)
N2—C12	1.546 (3)	C15—H15	0.98
N3—C22	1.327 (4)	C16—H16A	0.97
N3—C23	1.468 (4)	C16—H16B	0.97
N3—C25	1.472 (4)	C19—C20	1.528 (4)
C1—C9	1.412 (4)	C19—H19A	0.97
C1—C2	1.414 (4)	C19—H19B	0.97
C1—C6	1.425 (4)	C20—H20A	0.97
C2—C3	1.365 (4)	C20—H20B	0.97
C2—H2A	0.93	C17—C18	1.287 (5)
C3—C4	1.405 (5)	C17—H17	0.93
C4—C5	1.333 (5)	C18—H18A	0.93
C4—H4	0.93	C18—H18B	0.93
C5—C6	1.407 (5)	C21—C22	1.519 (4)
C5—H5	0.93	C21—H21A	0.97
C6—N1	1.363 (4)	C21—H21B	0.97
N1—C7	1.293 (4)	C23—C24	1.500 (6)
C7—C8	1.409 (4)	C23—H23A	0.97
C7—H7	0.93	C23—H23B	0.97
C8—C9	1.370 (4)	C24—H24A	0.96
C8—H8	0.93	C24—H24B	0.96
C9—C11	1.524 (4)	C24—H24C	0.96
C10—H10A	0.96	C25—C26	1.500 (5)
C10—H10B	0.96	C25—H25A	0.97
C10—H10C	0.96	C25—H25B	0.97
C11—C12	1.530 (4)	C26—H26A	0.96
C11—H11	0.98	C26—H26B	0.96
C12—C13	1.519 (4)	C26—H26C	0.96
C12—H12	0.98		
			
C3—O1—C10	118.6 (3)	C19—C14—H14	110.5
C11—O2—H2	109.5	C15—C14—H14	110.5
C21—N2—C16	109.7 (2)	C13—C14—H14	110.5
C21—N2—C20	107.1 (2)	C17—C15—C14	112.1 (3)
C16—N2—C20	105.7 (2)	C17—C15—C16	115.2 (3)
C21—N2—C12	115.5 (2)	C14—C15—C16	108.2 (3)
C16—N2—C12	107.4 (2)	C17—C15—H15	107.0
C20—N2—C12	111.0 (2)	C14—C15—H15	107.0
C22—N3—C23	118.2 (3)	C16—C15—H15	107.0
C22—N3—C25	125.2 (3)	N2—C16—C15	110.2 (3)
C23—N3—C25	116.6 (3)	N2—C16—H16A	109.6
C9—C1—C2	124.9 (3)	C15—C16—H16A	109.6
C9—C1—C6	117.1 (3)	N2—C16—H16B	109.6
C2—C1—C6	117.9 (3)	C15—C16—H16B	109.6
C3—C2—C1	120.6 (3)	H16A—C16—H16B	108.1
C3—C2—H2A	119.7	C14—C19—C20	109.3 (3)
C1—C2—H2A	119.7	C14—C19—H19A	109.8
O1—C3—C2	125.5 (3)	C20—C19—H19A	109.8
O1—C3—C4	114.3 (3)	C14—C19—H19B	109.8
C2—C3—C4	120.2 (3)	C20—C19—H19B	109.8
C5—C4—C3	121.1 (3)	H19A—C19—H19B	108.3
C5—C4—H4	119.5	C19—C20—N2	110.0 (2)
C3—C4—H4	119.5	C19—C20—H20A	109.7
C4—C5—C6	120.7 (3)	N2—C20—H20A	109.7
C4—C5—H5	119.6	C19—C20—H20B	109.7
C6—C5—H5	119.6	N2—C20—H20B	109.7
N1—C6—C5	116.6 (3)	H20A—C20—H20B	108.2
N1—C6—C1	123.9 (3)	C18—C17—C15	129.0 (4)
C5—C6—C1	119.5 (3)	C18—C17—H17	115.5
C7—N1—C6	116.3 (3)	C15—C17—H17	115.5
N1—C7—C8	125.1 (3)	C17—C18—H18A	120.0
N1—C7—H7	117.5	C17—C18—H18B	120.0
C8—C7—H7	117.5	H18A—C18—H18B	120.0
C9—C8—C7	119.5 (3)	N2—C21—C22	115.3 (2)
C9—C8—H8	120.2	N2—C21—H21A	108.4
C7—C8—H8	120.2	C22—C21—H21A	108.4
C8—C9—C1	118.1 (3)	N2—C21—H21B	108.4
C8—C9—C11	119.1 (3)	C22—C21—H21B	108.4
C1—C9—C11	122.9 (3)	H21A—C21—H21B	107.5
O1—C10—H10A	109.5	O3—C22—N3	122.5 (3)
O1—C10—H10B	109.5	O3—C22—C21	121.4 (3)
H10A—C10—H10B	109.5	N3—C22—C21	116.1 (3)
O1—C10—H10C	109.5	N3—C23—C24	112.9 (3)
H10A—C10—H10C	109.5	N3—C23—H23A	109.0
H10B—C10—H10C	109.5	C24—C23—H23A	109.0
O2—C11—C9	110.1 (2)	N3—C23—H23B	109.0
O2—C11—C12	110.0 (2)	C24—C23—H23B	109.0
C9—C11—C12	109.8 (2)	H23A—C23—H23B	107.8
O2—C11—H11	109.0	C23—C24—H24A	109.5
C9—C11—H11	109.0	C23—C24—H24B	109.5
C12—C11—H11	109.0	H24A—C24—H24B	109.5
C13—C12—C11	113.6 (3)	C23—C24—H24C	109.5
C13—C12—N2	107.7 (2)	H24A—C24—H24C	109.5
C11—C12—N2	114.0 (2)	H24B—C24—H24C	109.5
C13—C12—H12	107.0	N3—C25—C26	112.5 (3)
C11—C12—H12	107.0	N3—C25—H25A	109.1
N2—C12—H12	107.0	C26—C25—H25A	109.1
C12—C13—C14	109.9 (2)	N3—C25—H25B	109.1
C12—C13—H13A	109.7	C26—C25—H25B	109.1
C14—C13—H13A	109.7	H25A—C25—H25B	107.8
C12—C13—H13B	109.7	C25—C26—H26A	109.5
C14—C13—H13B	109.7	C25—C26—H26B	109.5
H13A—C13—H13B	108.2	H26A—C26—H26B	109.5
C19—C14—C15	109.1 (3)	C25—C26—H26C	109.5
C19—C14—C13	106.5 (3)	H26A—C26—H26C	109.5
C15—C14—C13	109.7 (3)	H26B—C26—H26C	109.5
			
C9—C1—C2—C3	−178.8 (3)	C16—N2—C12—C11	−160.6 (2)
C6—C1—C2—C3	−1.0 (5)	C20—N2—C12—C11	84.3 (3)
C10—O1—C3—C2	9.8 (6)	C11—C12—C13—C14	−150.9 (3)
C10—O1—C3—C4	−170.9 (4)	N2—C12—C13—C14	−23.6 (3)
C1—C2—C3—O1	−179.6 (3)	C12—C13—C14—C19	74.2 (3)
C1—C2—C3—C4	1.0 (5)	C12—C13—C14—C15	−43.7 (4)
O1—C3—C4—C5	−179.7 (4)	C19—C14—C15—C17	−176.2 (3)
C2—C3—C4—C5	−0.3 (6)	C13—C14—C15—C17	−59.8 (4)
C3—C4—C5—C6	−0.5 (6)	C19—C14—C15—C16	−48.0 (3)
C4—C5—C6—N1	−179.7 (4)	C13—C14—C15—C16	68.3 (3)
C4—C5—C6—C1	0.5 (5)	C21—N2—C16—C15	−173.8 (2)
C9—C1—C6—N1	−1.6 (5)	C20—N2—C16—C15	71.1 (3)
C2—C1—C6—N1	−179.6 (3)	C12—N2—C16—C15	−47.5 (3)
C9—C1—C6—C5	178.2 (3)	C17—C15—C16—N2	107.2 (3)
C2—C1—C6—C5	0.2 (4)	C14—C15—C16—N2	−19.1 (3)
C5—C6—N1—C7	−178.2 (3)	C15—C14—C19—C20	67.4 (4)
C1—C6—N1—C7	1.6 (5)	C13—C14—C19—C20	−50.9 (4)
C6—N1—C7—C8	0.1 (5)	C14—C19—C20—N2	−14.0 (4)
N1—C7—C8—C9	−1.8 (5)	C21—N2—C20—C19	−168.1 (3)
C7—C8—C9—C1	1.7 (5)	C16—N2—C20—C19	−51.2 (3)
C7—C8—C9—C11	−179.1 (3)	C12—N2—C20—C19	64.9 (3)
C2—C1—C9—C8	177.7 (3)	C14—C15—C17—C18	133.4 (5)
C6—C1—C9—C8	−0.1 (4)	C16—C15—C17—C18	9.1 (6)
C2—C1—C9—C11	−1.5 (5)	C16—N2—C21—C22	66.6 (3)
C6—C1—C9—C11	−179.4 (3)	C20—N2—C21—C22	−179.1 (3)
C8—C9—C11—O2	−9.1 (4)	C12—N2—C21—C22	−55.0 (3)
C1—C9—C11—O2	170.2 (3)	C23—N3—C22—O3	3.5 (5)
C8—C9—C11—C12	112.2 (3)	C25—N3—C22—O3	−174.3 (3)
C1—C9—C11—C12	−68.6 (4)	C23—N3—C22—C21	−174.8 (3)
O2—C11—C12—C13	70.3 (3)	C25—N3—C22—C21	7.4 (5)
C9—C11—C12—C13	−51.0 (3)	N2—C21—C22—O3	3.3 (4)
O2—C11—C12—N2	−53.6 (3)	N2—C21—C22—N3	−178.4 (3)
C9—C11—C12—N2	−175.0 (2)	C22—N3—C23—C24	86.3 (4)
C21—N2—C12—C13	−164.9 (2)	C25—N3—C23—C24	−95.7 (4)
C16—N2—C12—C13	72.3 (3)	C22—N3—C25—C26	96.8 (4)
C20—N2—C12—C13	−42.8 (3)	C23—N3—C25—C26	−81.1 (4)
C21—N2—C12—C11	−37.8 (3)		

### Hydrogen-bond geometry (Å, °)

**Table:**

*D*—H···*A*	*D*—H	H···*A*	*D*···*A*	*D*—H···*A*
O2—H2···Cl1	0.82	2.25	3.038 (2)	161
C19—H19A···Cl1^i^	0.97	2.70	3.580 (4)	150
C20—H20B···O2	0.97	2.33	3.001 (4)	126
C21—H21B···Cl1	0.97	2.76	3.650 (3)	152
C21—H21B···O2	0.97	2.58	3.169 (4)	119

Symmetry codes: (i) *x*+1, *y*, *z*.
